# The future of health digitalization: The case of Primary Health Care in Portugal

**DOI:** 10.1093/eurpub/ckac129.200

**Published:** 2022-10-25

**Authors:** M Peyroteo dos Santos, L Lapao

**Affiliations:** NOVA, Lisbon, Portugal

## Abstract

**Background:**

The sustainability of Healthcare Services will depend on the proper implementation of the digital transformation of Primary Health Care (PHC) Services. COVID-19 Pandemic have shown precisely this, where there was pressure from health Professionals and managers for the integration of teleconsultations within the health work processes. This study aims at developing scenarios for the digitalization of PHC in the Portuguese Health System.

**Methods:**

This study follows the methodology of scenario development, similar to the future of community pharmacy services scenario study. A conceptual model of PHC services was developed, based on the literature and a set of interviews. The target group was the primary healthcare professionals: family doctors, nurses, and operational assistants. The time horizon was 2032 and, by selecting actors from different regions of Portugal, it was possible to achieve broad representativeness.

**Results:**

Three focus groups were conducted. The first enabled to identify the two driving forces that may influence the digitalization of PHCs in the next 10 years: 1) service innovation and 2) Governance and Regulations. These two driving forces enabled to design three plausible scenarios: a) Innovate or Fade-away; b) Isolated PHC and c) Digital PHC. These scenarios were developed, and their impacts were reflected upon. It was found that the role of human Resources is critical.

**Conclusions:**

The process of reflection and discussion for the identification of the different driving forces made the different actors discuss the different points of view and find a meeting point to reach a conclusion. Furthermore, this study allowed the different stakeholders to understand the measures and actions to be taken for PHC digitization to be implemented in the most effective way, allowing the sustainability of the National Health Service in Portugal, which until this point were only ideas discussed at the institutional and individual level.

